# Professor Koch on Milk Tuberculosis

**Published:** 1901-08-03

**Authors:** 


### The Hospital

A Journal of

The Medical Sciences and Hospital Administration.

Vol. XXX.—No. 775.] Edited by Sir Henry Burdett, K.C.B., and Solomon C. Smith, M.D., M.R.C.P. [August 3, 1901.

### Professor Koch on Milk Tuberculosis.

So far as the Congress on Tuberculosis which was held last week was intended to draw public attention to the great subject which its promoters had at heart there is no doubt that it succeeded in perfection. There was something so dramatic in the sudden overturning by Professor Koch of the very notion which, as far as the world knew, he had come to bless, that public interest was at once aroused. With a programme so framed as to emphasise in every way the necessity of adopting stringent measures for preventing the sale of tuberculous meat and milk, Professor Koch opened the ball by a declaration that bovine and human tuberculosis are different affairs, and that infection by milk and meat is so rare that he does not deem it advisable to take any measures against it. Needless to say that the butchers and the milkmen were at once thrown into a state of jubilation by this pronouncement, and that many scornful things have been said as to the untrustworthy nature of medical dogmas. And we cannot but think that a good many of these scornful things are well deserved, and that the medical profession, or such part of it as has devoted itself of late years to the dissemination of popular pathology on this subject, may well spend its autumn holiday eating humble pie.

For the last few years we have lived in the midst of a newspaper crusade against tuberculosis. Everything has been sacrificed to simplicity of view. Infection has been made a fetish; no sooner was it shown that tuberculosis was infective, and that tubercle bacilli were to be found in milk, than it became "obvious"—dreadful word—that we were exposed to endless dangers from consuming such infected material.

Perhaps if we had not crusaded quite so much we might have had more time for logic. Perhaps if we had had less respect for authority we might have listened to those in our midst who have never ceased to say, just as Professor Koch says now, that the infection of human beings by milk is but a very rare occurrence. Let us recall that, putting mere hypothesis on one side, the proof of infection by milk depends almost entirely upon a statistical argument. The mortality from tuberculosis in its various forms has diminished greatly during the last fifty years, if we take all ages together; but we are told that it has not only not diminished, but has actually increased to the tune of 27·7 per cent. if we take only the first year of life into consideration. Moreover, we find during this first year of life an enormous preponderance of cases of *tabes mesenterica*, which is "the form of tuberculosis most identified with the reception of the tubercular infection into the digestive tract," and if this excess of intestinal and mesenteric disease is due to milk-drinking of course the condemnation of the milk is complete. The weak spot in the argument is that it is assumed that all these babies who are returned as dying of *tabes mesenterica* did actually die of tuberculosis, as, of course, they ought to have done according to the official "Nomenclature of Diseases." Professor Koch has now stated publicly and with great *éclat* that, notwithstanding the enormous consumption of milk known to contain bovine tuberculosis, the number of children shown by *post-mortem* examination to have been affected with primary tuberculosis of the intestines is very small, and with our usual respect for authority we now listen to this teaching. But it has been taught all along.

All honour to Professor Koch if he should turn out to be correct in his explanation of the facts, but as to the facts, are they not written in the records of the *post-mortem* rooms? And as to the teaching, has it not been constantly held by those who chose to stand against the stream of popularised pathology, that there was no just ground for the assertion that all these *tabes mesenterica* babies were affected with primary intestinal tuberculosis?

In a paper read in 1894 before the Medical Society Dr. Walter Carr pointed out that the tuberculous disease which occurs in children starts much more frequently in the thorax than in the abdomen. Dr. Still, speaking before the British Medical Association in 1899, demonstrated the same thing, and again in the *Practitioner* of last month gave statistics from the dead-house which showed that, plausible as is the milk theory, "the facts of the *post-mortem* room are overwhelmingly opposed to it." Dr. Maguire also has written in much the same sense, showing that *tabes mesenterica*, largely as it figures in statistics, is practically unknown as a cause of death in young children. Truly these investigators did not draw the distinction which has now been drawn between bovine and human tuberculosis, but they did show that we were on the wrong track in attributing so much to milk infection.

If we had not all gone a-crusading perhaps we might have listened to the prophets in our midst who pointed out how weak was the foundation on which all this superstructure of milk infection had been erected. Great, however, is the power of authority, and we had to wait until one even greater came from Germany to prick the bubble of our content.

